# Perspectives of primary care physicians in Spain on malaria: a cross-sectional survey and retrospective review of cases

**DOI:** 10.1186/s12936-023-04826-6

**Published:** 2024-01-04

**Authors:** Manuel Linares-Rufo, Harold Bermudez-Marval, Carlos García-Bertolín, Joaquín Santos-Galilea, Javier Balsa-Vázquez, Ramón Pérez-Tanoira, Laura Santos-Larrégola, Juan Cuadros-González, Gerardo Rojo-Marcos, José-Manuel Ramos-Rincón

**Affiliations:** 1 Microbiology Department, Hospital Universitario Príncipe de Asturias, Alcalá de Henares, Madrid Spain; 2 Foundation iO, Madrid, Spain; 3 Internal Medicine Department, Hospital Universitario Príncipe de Asturias, Alcalá de Henares, Madrid Spain; 4 Biomedicine and Biotechnology Department, University Alcalá de Henares, Alcalá de Henares, Madrid Spain; 5 Clinical Medicine Department, Miguel Hernández of Elche University, Elche, Alicante Spain; 6 Internal Medicine Department, Dr. Balmis General University Hospital, Alicante Institute for Health and Biomedical Research (ISABIAL), Alicante, Spain

**Keywords:** Survey, Malaria, Immigrant, Spain, Physicians, Primary care

## Abstract

**Background:**

In Spain, the risk of imported malaria has increased in recent years due to the rise in international travel and migration. Little is known about the knowledge, information sources, clinical practice, and specific needs of primary care physicians (PCPs) concerning malaria despite the pivotal role played by these professionals in managing the health of tourists. The objective of this study was to assess the knowledge, attitudes, and practices of PCPs in Spain regarding malaria.

**Methods:**

This research analyses data from (1) a cross-sectional nationwide survey assessing the knowledge and attitudes of PCPs regarding malaria, and (2) a retrospective review of 373 malaria cases appearing in primary care medical records (PCMRs) in the Madrid area over the past 15 years to determine how cases were documented, managed, or characterized in the primary care setting.

**Results:**

The survey findings reveal a modest level of self-perceived familiarity with malaria (221/360, 57.6%), even though 32.8% of the practitioners reported having delivered care for confirmed or suspected cases of the disease, these practitioners had greater knowledge of malaria (80.4%) compared to physicians who reported not having delivered care for malaria (19.6%, p < 0.001). Ten percent of the survey participants did not know the name of the mosquito that transmits malaria, and only 40.7% would promptly request malaria testing for a traveller with symptoms after a trip to an endemic area. Responses provided by younger PCPs varied to a greater extent than those of their more experienced colleagues regarding prevention practices and patient management.

A review of PCMRs showed that only 65% of all patients were recorded as such. Among those registered, only 40.3% had a documented malaria episode, and of those, only 16.6% received proper follow-up. Only 23.7% of the patients with a PCMR had a record that specifically indicated travel to an endemic country or travel classified as visiting friends and relatives (VFR).

**Conclusions:**

The findings of this study underscore the critical role of PCPs in the field of travel medicine, particularly given the increase in imported malaria cases. These results highlight the need for targeted training in travel medicine and the need to ensure optimal patient education in care settings.

## Background

International travel has increased substantially in recent decades, with cross-border tourism growing by 4% in 2019, reaching 1.5 billion travellers. Post-pandemic recovery data show that the number of foreign tourists climbed to 80% of pre-pandemic levels in the first quarter of 2023 [1, 2]. In an era of globalization, travel-related illnesses have become a focus of public health concern, especially in Europe, where travel health services are well-established. A recent review estimated that between 43 and 79% of travellers who visited developing nations became ill [3].

A key health concern for travellers is malaria, a leading cause of morbidity and mortality worldwide, particularly in tropical and subtropical regions. Despite advancements in prevention and treatment, malaria continues to impose a substantial burden on public health, with millions of cases and a high mortality rate each year [4].

Commonly, primary care physicians (PCPs) are the first line of contact for pre-travel consultation, with up to 65% of travellers seeking advice from their general practitioner; furthermore, PCPs are a preferred source of care for travellers returning from endemic countries [5, 6]. Given their comprehensive training, particularly in prevention and counseling, they are ideally positioned to provide adequate care for the large number of travellers [7].

PCPs play a crucial role in managing tourists, including older travellers [8–11]. They routinely see patients with comorbidities such as diabetes, hypertension, or kidney failure, many of whom also travel for pleasure [7, 12]. With regard to malaria, their responsibilities encompass diagnostic suspicion, early detection, referral to a specialist care facility, and primary prevention (i.e., education on preventive measures). However, the effectiveness of these interventions largely depends on the depth of knowledge and competence of primary care professionals. Moreover, care delays can pose a risk for more severe disease [13].

Current data available concerning the knowledge, information sources, clinical practices, and the needs of these physicians is limited. Several worldwide studies on PCP knowledge of travel medicine (TM) contain significantly disparate findings, with some reporting better results [14, 15] while others report worse results [16–18] depending on the country where the study was conducted.

In Spain, malaria is a comparatively rare infection. Between 2019 and 2021, a total of 1482 cases were reported, of which 1477 (99.7%) were confirmed [19, 20]. Hence, it is crucial to assess the preparedness and knowledge of PCPs regarding malaria and identify potential gaps in their training and performance. Therefore, the objectives of this study were (1) to investigate the knowledge and clinical practices of PCPs in relation to malaria, and (2) to examine malaria management within primary care and review actions taken in a specific healthcare region in Spain over the past 15 years. This study thus seeks to (a) evaluate the level of proficiency among PCPs in Spain in terms of malaria chemoprophylaxis, and (b) assess PCPs adherence to protocols and guidelines related to the management of malaria cases.

## Methods

To accomplish these objectives, we conducted 2 studies: (1) a cross-sectional survey aimed at assessing the knowledge and attitudes of PCPs regarding malaria and (2) a retrospective chart review of 373 malaria cases reported in primary care medical records (PCMRs).

### Survey

This observational survey-based cross-sectional study consisting of an online questionnaire administered to PCPs practicing in primary healthcare centres in Spain was carried out from July 1 to July 31, 2023. The meticulously designed online survey comprises 14 multiple-choice questions concerning the management of malaria in primary care [21, 22]. The items were drafted by ML and revised following feedback and validation from 25 expert PCPs.

Standardized questionnaires were distributed to a random sample of 2500 PCPs practicing in Spain who belong to any of the 3 major Spanish primary care scientific societies (i.e., SEMERGEN, SEMFYC, SEMG). Participants were requested to complete the survey and were explained that participation was voluntary and their responses would be anonymous. The survey was administered through an online platform accessible to PCPs, with strict attention paid to safeguarding participant confidentiality and anonymity. Participants received a URL granting access to the survey and all relevant information such as the aim and importance of the study, the voluntary nature of participation, data security, and anonymity.

The number of respondents that made up the final sample (n = 387) is considered representative of the total population of 29,000 PCPs in Spain, with a 95% confidence interval and a 5% margin of error, and is consistent with other studies involving similar populations [23] No formal sample size calculation was performed. The sample size was defined as the total number of PCPs who responded to the questionnaire.

### PCMR review

A retrospective chart review was conducted on 373 cases of patients diagnosed with malaria who underwent treatment between 2005 and 2022 in the Príncipe de Asturias University Hospital, located in the city of Alcalá de Henares, near metropolitan Madrid. Of note, the PCMR and hospital medical records operate independently. The analysis focused on identifying all entries made by PCPs in the PCMR, particularly related to the management and treatment of cases.

### Data analysis

Categorical variables were expressed as frequencies (percentages) and continuous variables as median values (interquartile range). The response rate was analysed according to years of professional practice (< 15 vs. ≥ 15). Differences in frequency were analysed by using Chi-square or Fisher’s exact test.

The data retrieved from the PCMR were analysed descriptively, with categorical variables expressed as frequencies (percentages). The characteristics of malaria episodes were analysed with and without the PCMR. Frequency differences were assessed using the Chi-square or Fisher’s exact test, with statistical significance set at p < 0.05. Data were managed using SPSS v20.0 (IBM Corp., Armonk, NY, USA).

### Ethical considerations

Completion of the survey was interpreted as an indication of informed consent to the anonymous use of the data. Participants received no funding or other incentives to take part. It is important to note that the confidentiality and anonymity of all data collected were rigorously safeguarded. Approval for this study was obtained from the Príncipe de Asturias University Hospital (EXP: OE 07/2022). The study was conducted in full compliance with standard ethical principles and applicable regulations.

## Results

### Survey

A total of 3990 emails containing links to the survey were sent, and 387 PCPs responded (response rate, 9.67%). The median length of professional experience was 25 years (P25: 15; P75: 31 years). Table 1 presents a breakdown of survey responses, comparing professional experience of < 15 years with ≥ 15 years.

**Table 1:** Malaria survey responses among primary care physicians

Question	All (N = 360) n (%)	< 15 years of professional practice (N = 83) n (%)	≥ 15 years of professional practice (N = 277) n (%)	P value
Have you had any suspected or confirmed cases of malaria in your practice?				
No	260 (67.2)	61 (67.8)	199 (67.0)	**0.891**
Yes	127 (32.8)	29 (32.2)	88 (33.0)	
How would you rate your knowledge of malaria? (n = 384)				
High	22 (5.7)	4 (4.4)	18 (6.1)	0.837
Low	221 (57.6)	50 (55.6)	171 (58.2)	
Medium	136 (35.49)	50 (55.6)	171 (58.2)	
Null	5 (1.3)	1 (1.1)	4 (1.4)	
Which of the following mosquitoes causes malaria?				
*Aedes*	39 (10.1)	12 (13.3)	27 (9.2)	0.477
*Anopheles*	345 (89.5)	78 (86.7)	267 (90.5)	
*Culex*	1 (0.3)	0 (0.0)	1 (0.3)	
What is your course of action when travelers to malaria-endemic countries come to your practice?				
I manage part and refer the rest to specialists	151 (39.8)	42 (48.8)	109 (37.2)	0.070
I refer them to tropical medicine units	173 (45.6)	30 (34.9)	143 (48.8)	
I manage these patients in my regular practice	55 (14.5)	14 (16.3)	41 (14.0)	
Which of these travelers do you consider to be at the highest risk of contracting malaria?				
Elderly	28 (7.3)	4 (84.4)	24 (8.2)	0.227
Pregnant	134 (34.9)	39 (43.3)	95 (32.3)	
Child	24 (6.3)	5 (5.6)	19 (6.5)	
VFR	198 (51.6)	42 (46.7)	156 (53.1)	
When faced with a patient returning from a malaria-endemic area, which of the following symptoms do you consider most significant?				
Headache	3 (0.8)	2 (2.2)	1 (0.3)	0.002
Diarrhea	8 (2.1)	5 (5.6)	3 (1.0)	
Fever	78 (20.2)	17 (19.1)	61 (20.5)	
All of the above	295 (76.5)	63 (70.8)	232 (78.1)	
Regarding the prevention of mosquito bites, which of the following measures do you consider most appropriate?				
Mosquito net	2 (0.5)	1 (1.1)	1 (0.3)	0.030
Repellents	5 (1.3)	0 (0.0)	5 (1.7)	
Appropriate clothing	1 (0.5)	2 (2.2)	0 (0.0)	
All the above are valid	376 (97.7)	86 (96.6)	290 (98.0)	
What sources of information do you consult regularly?				
Clinical guidelines of scientific societies	201 (52.5)	43 (47.8)	158 (53.9)	0.369
Spanish Ministry of Health	109 (28.5)	27 (30.0)	82 (28.0)	
Scientific journals/PubMed	42 (11.0)	14 (15.6)	28 (9.6)	
Search engines (e.g., Google)/generative artificial intelligence tools (e.g., ChatGPT)	31 (8.19)	6 (6.7)	25 (8.5)	
Which of the following pieces of advice do you think is most appropriate to give to a traveler visiting a malaria-endemic area?				
Antimalarial chemoprophylaxis	267 (69.2)	56 (62.2)	211 (71.3)	0.023
Use of insect repellent and appropriate clothing	84 (21.8)	29 (32.2)	55 (18.6)	
Vaccination	23 (6.0)	2 (2.2)	21 (7.1)	
General advice (e.g., water, food, sunscreen, travel insurance)	12 (3.19)	9 (3.0)	9 (3.0)	
How is malaria diagnosed in your area?				
Thick blood smear	148 (41.1)	40 (48.2)	108 (39.0)	0.541
Antigen test	10 (2.8)	1 (1.2)	9 (3.2)	
Polymerase chain reaction (PCR)	49 (13.6)	16 (19.3)	33 (11.9)	
All of the above	153 (42.5)	26 (31.3)	127 (45.8)	
In the case of a patient returning from a tropical area with fever, diarrhea, and general malaise, what do you consider the most appropriate management?				
Request full battery of laboratory tests and monitor general condition	225 (58.3)	53 (58.9)	172 (58.1)	0.041
Refer the patient to the emergency department to rule out malaria	158 (40.7)	37 (41.1)	120 (40.5)	
Daily telephone monitoring	4 (1.0)	0 (0.0)	4 (1.4)	

In response to the question “Have you ever encountered suspected or confirmed cases of malaria in your practice?”, 32.8% responded affirmatively, with no significant differences based on years of professional experience. When asked to rate their knowledge of malaria, 57.6% described it as low, while 35.4% considered it moderate, and 5.7% high, with no notable variations based on length of experience. Of the practitioners with high knowledge, 68.2% had encountered suspected or confirmed cases of malaria, while 31.8% had not (p = 0.009). Among those with low knowledge, 19.6% had encountered such cases, and 80.4% had not (p < 0.001). For those with medium knowledge, half had encountered suspected or confirmed cases of malaria, while the other half had not. When prompted to answer the question “Which type of mosquito is responsible for transmitting malaria? (*Aedes*, *Anopheles*, or *Culex*)”, 89.5% correctly identified “*Anopheles*” regardless of experience.

Regarding travellers to malaria-endemic regions seen in clinical practice and the preventive measures taken with these patients, 45.6% of the PCPs indicated that they refer these individuals to preventive medicine units. Notably, 34.9% of physicians with fewer than 15 years of experience did so, compared to 48.8% of their more experienced colleagues (p = 0.07). Furthermore, 39.9% of physicians manage a portion of these travellers and refer the rest to preventive medicine units. In this case, those with fewer years of experience adopted this approach more frequently (48.8% vs. 37.2%) than their more veteran counterparts (p = 0.07). A mere 14.5% provide care for all such patients within their regular practice.

For the question “Which of these travellers (elderly, pregnant woman, child, or VFR) do you consider to be at the highest risk of contracting malaria?”, 51.6% selected VFRs, 34.9% answered pregnant women, 7.3% the elderly, and 6.3% children. There were no significant differences in terms of experience.

In response to a question concerning patients who return from a malaria-endemic area (i.e., “Which of the following symptoms (headache, diarrhea, fever, sweating, or all) do you consider most important?”), 76.5% identified all of them, while fever was the response given by 20.2%.

Regarding mosquito bite prevention, when asked “Which of the following measures do you consider most appropriate (mosquito nets, repellents, appropriate clothing, or are all of the above)?”, 97.7% stated that all were valid.

Responses to the question “What sources of information do you regularly consult?” revealed that 52.5% referred to guidelines from scientific societies, 28.5% consulted information from the Spanish Ministry of Health, 11% relied on scientific journals through PubMed, and 8.1% used Google or ChatGPT. There were no significant differences based on years of professional experience.

In response to the question “Which of these pieces of advice do you consider most suitable to give to a traveller visiting a malaria-endemic area?”, 69.2% recommended antimalarial prophylaxis. Younger professionals were less likely to provide this response than those with more experience (62.2% vs. 71.3%; p = 0.023). Additionally, 21.8% suggested using insect repellent as the primary preventive measure, with younger professionals choosing this option more often (32.2% vs. 18.6%; p = 0.023). A 6.0% minority advocated for vaccination, while 3.1% provided general advice on water and food. No differences were found when the data were analysed in terms of years of experience; however, these responses are clearly incorrect.

When asked how malaria is diagnosed in their area, 42.5% stated that all available methods are used (i.e., thick blood smear, PCR, antigen tests). Interestingly, this response was more frequent among more experienced respondents (43.3% vs. 32.3%; p = 0.02). Additionally, 41.4% selected the thick blood smear as the preferred diagnostic method, and more experienced physicians selected this option less frequently (39.0% vs. 48.2%; p = 0.043).

### PCMR review

**Fig. 1:**
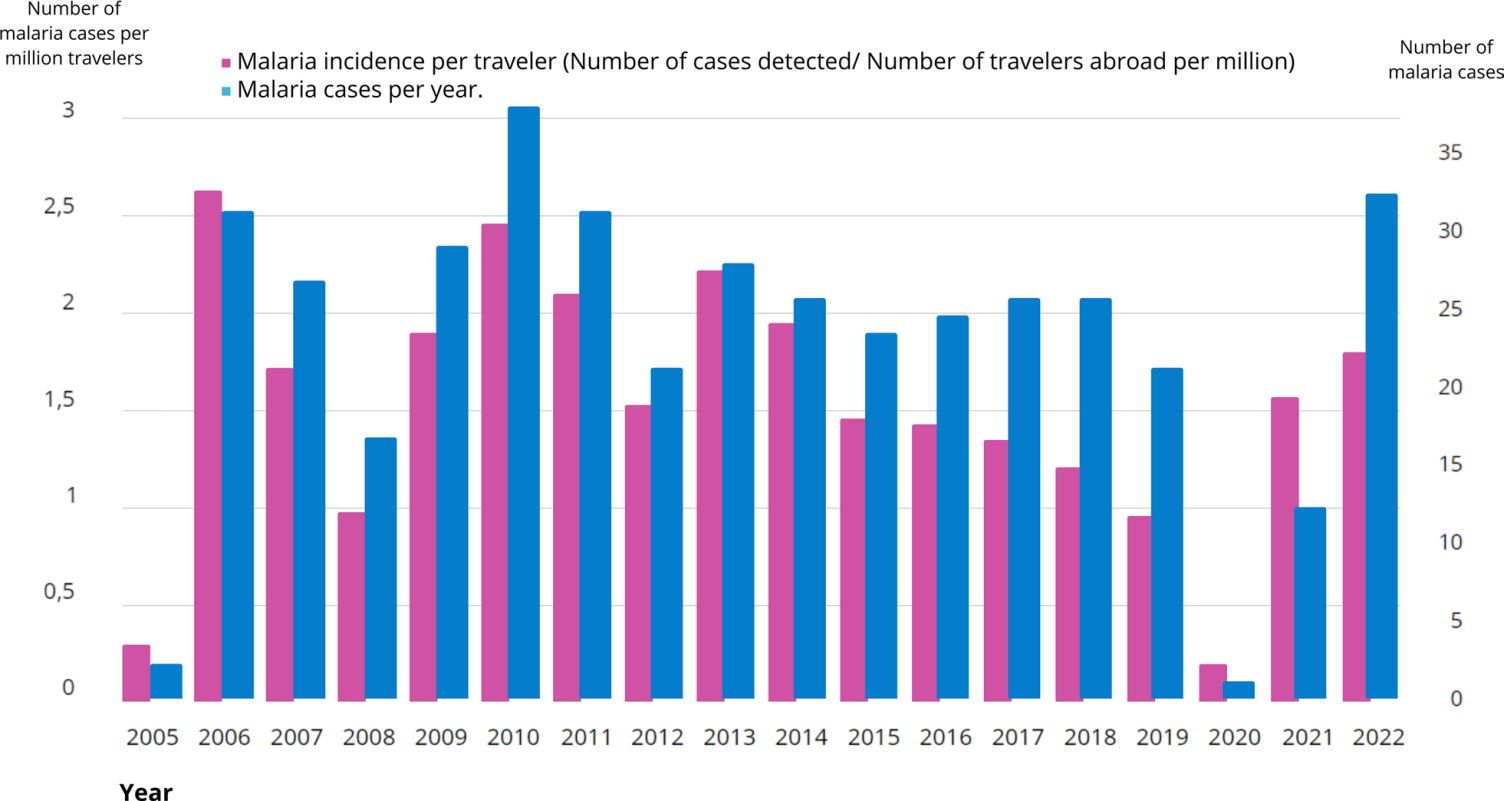
Malaria cases per year and incidence per million travellers

A total of 373 cases of malaria were diagnosed from 2005 to 2022. The average yearly number of diagnosed cases exceeds 20, with a notable decline in 2020 and 2021 due to the pandemic and related travel restrictions. Figure 1 shows the number of malaria cases per year and malaria incidence per million travellers.

Among the 253 patients with a PCMR who were subsequently diagnosed with malaria in the course of a hospital visit, a specific malaria episode was entered in the PCMR for 102 individuals (40.3%).

**Fig. 2:**
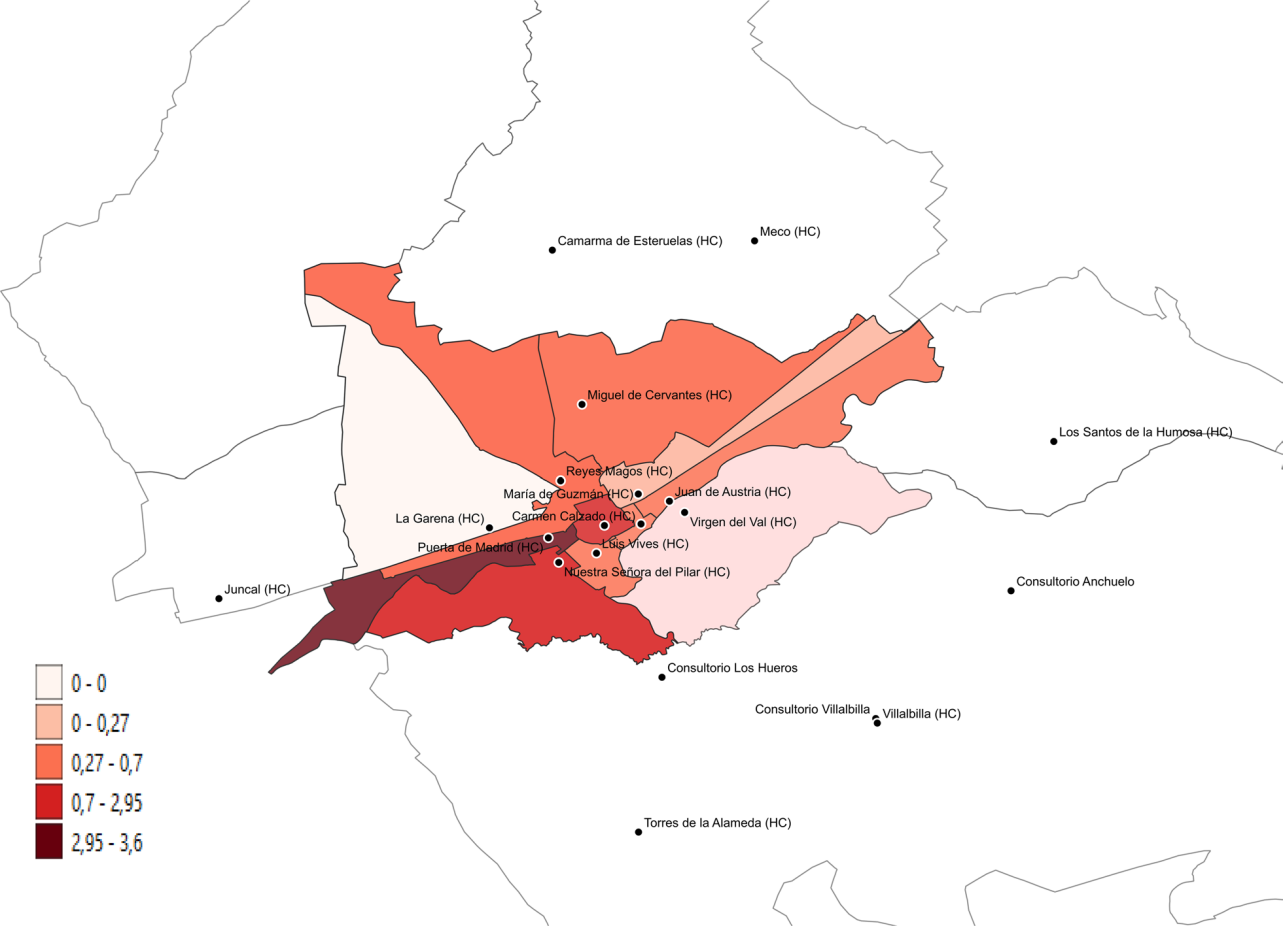
Map of malaria incidence per 1000 inhabitants in a health area in the Madrid region by primary care centre

An examination of malaria cases by basic healthcare area (geolocation of their assigned primary care facility) allowed us to create an incidence map of cases (Fig. 2 and Tables 2, 3).

**Table 2:** Health area population and cases

Area served by health center	Population	Average income	Percentage of foreign-born residents	Malaria cases	Malaria incidence (per 10,000 inhabitants)
Carmen Calzado	15,581	36,473.91	20.29%	13	0.8343
Juan de Austria	14,753	34,626.36	20.72%	10	0.6778
La Garena	8386	52,597.25	9.73%	0	0
Luis Vives	24,386	35,749.13	14.57%	16	0.6561
Manuel Merino	11,560	32,889.11	18.02%	8	0.692
María de Guzmán	22,201	41,897.53	17.53%	6	0.2702
Miguel de Cervantes	27,256	44,702.77	6.95%	19	0.6971
Nuestra Señora del Pilar	17,646	27,909.00	21.78%	52	2.9468
Puerta de Madrid	13,335	27,869.70	18.76%	48	3.5995
Reyes Magos	26,406	36,167.13	19.08%	15	0.5681

**Table 3:** Characteristics of diagnosed episodes of malaria recorded in or missing from the primary care medical records

Variables	All (N = 373) n (%)	Without record in primary care (N = 120) n (%)	With record in primary care (N = 253) n (%)	P
Age				
Median age (IQR)	33 (23–32)	30 (18–38)	35 (25–46)	0.001
Age group				0.467
0–14 years	52 (13.9)	19 (15.8)	33 (13.0)	
≥ 15 years	321 (86.1)	101 (84.2)	220 (87.0)	
Sex				0.230
Female	194 (52.0)	57 (47.5)	137 (54.2)	
Male	179 (48.0)	63 (52.5)	116 (45.8)	
Type of patient				
Native of Europe	15 (4.0)	1 (0.5)	14 (7.8)	0.404
Second-generation immigrant	25 (6.7)	9 (4.6)	16 (8.9)	0.995
Immigrant with extended residence in Spain	231 (61.9)	136 (70.1)	95 (53.1)	< 0.001
Recent immigrant	56 (15.0)	26 (13.4)	30 (16.8)	0.174
Health tourist	12 (3.2)	7 (36.6)	5 (2.8)	0.060
Tourist	21 (5.6)	10 (5.2)	11 (6.1)	0.002
Not available	13 (3.5)	5 (2.6)	8 (4.4)	-
Country of birth				
Equatorial Guinea	238 (63.8)	78 (65.0)	160 (63.2)	0.851
Spain	41 (11.0)	9 (7.5)	32 (12.6)	0.138
Ghana	8 (2.1)	3 (2.5)	5 (2.0)	1.00
Nigeria	61 (14.4)	20 (16.7)	41 (12.2)	0.563
Mali	5 (1.3)	1 (0.8)	4 (1.6)	1.0
Other African countries	15	6	9	-
Other European countries	4	2	2	-
Americas	1	1	0	-
History of prophylaxis				
No	241 (64.6)	75 (62.5)	166 (65.6)	0.556
Yes, complete	11 (4.3)	0 (0.0)	11 (2.9)	0.020
Yes, incomplete	33 (8.8)	4 (3.3)	29 (11.5)	0.010
Migrant	39 (10.5)	20 (16.7)	19 (7.5)	0.007
No data	49 (13.3)	21 (17.5)	28 (11.1)	0.086
HIV serology test				< 0.001
Negative	193 (51.7)	45 (37.5)	148 (58.5)	
Positive	46 (11.7)	14 (11.7)	32 (12.6)	
No data	134 (35.9)	61 (50.8)	73 (28.9)	

Other African countries: Congo (n = 5), Ivory Coast (n = 2), Senegal (n = 2), Benin (n = 1), Cameroon (n = 1), Chad (n = 1), Guinea (n = 1), Sierra Leone (n = 1), Sudan (n = 1). Other European countries: France (n = 1), Netherlands (n = 1), Poland (n = 1), UK (n = 1). The Americas: Guyana (n = 1),

Finally, records of drugs prescribed for malaria chemoprophylaxis (at any date/time) within the primary care history of patients who had a prior primary care record were analysed. Additionally, entries concerning 3 vaccines (hepatitis A, hepatitis B, and typhoid fever) recommended for individuals travelling to high-risk countries were examined. The results are presented in Table 4.

**Table 4:** Record of chemoprophylaxis prescriptions and vaccinations in the primary care medical record

Variables	All (N = 253) n (%)
Episode created in the medical record based on travel to endemic countries	
No	193 (76.3)
Yes	60 (23.7)
Episode of malaria created	
No	151 (59.7)
Yes	102 (40.3)
Follow-up in primary care	
No	211 (83.4)
Yes	42 (16.6)
Antimalarial prescription	
No	217 (85.8)
Yes	36 (14.2)
Prescription of antimalarials after travel	
No	186 (73.5)
Yes	67 (26.5)
Hepatitis B vaccination	
No	195 (77.1)
Yes	58 (22.9)
Hepatitis A vaccination	
No	220 (87.0)
Yes	33 (13)
Typhoid vaccination	
No	225 (89.0)
Yes	28 (11.0)
HIV	
No	6 (2.4)
Yes	26 (10.3)
Missing	221 (87.4)

## Discussion

Based on the results of the present study, a number of conclusions can be drawn regarding the knowledge and practices associated with malaria management within the primary care setting. These findings provide valuable insights that can guide the development of strategies to enhance training and interventions in malaria management within primary care.

Data from this study indicate that there is an urgent need to provide education and guidance to healthcare professionals on malaria prevention, recognizing warning signs, and the significance of seeking early medical attention. Timely detection of malaria is crucial for efficient disease management, and PCPs should undergo training to equip them with the necessary skills to effectively handle the disease.

### Malaria training in primary care

In Spain, PCPs play a pivotal role in delivering TM advice. The study aimed to shed light on the frequency and nature of TM consultations, encompassing both clinical and educational components [24, 25]. Notably, there is a high demand for concise, up-to-date travel advice among practitioners.

The findings reveal that a considerable proportion of the general practitioners surveyed did not adhere to recommended guidelines on malaria management. Use of standardized, regularly updated, and easily accessible sources of travel advice for GPs could mitigate uncertainties for both providers and patients.

Continuous education and training programs conducted by experienced professionals are strongly advocated for all PCPs. This proactive approach is crucial to bridge the knowledge gap in malaria and TM, ensuring that practitioners are prepared to offer the best care to their patients.

Few studies have investigated primary healthcare provider-related barriers to the provision of pre-travel health advice, particularly to VFRs [26, 27]. The data suggest that fostering networking between doctors and referral centres for malaria would bolster the use of best practices concerning chemoprophylaxis and protection against mosquito bites, especially targeting at risk patients.

Beneath this lack of studies lie several contributing factors, including the influence of individuals in transit, temporary migrants, and undocumented migrants. This fact presents an opportune moment to assess and address the existing gaps, potentially initiating a fresh approach to enhance communication effectively [28].

### Under-recording of malaria and HIV episodes

The study reveals a significant issue by which episodes of malaria and cases of HIV infection are often absent from PCMRs. Nearly 3 in 5 patients with malaria and 1 in 5 HIV who seek treatment in primary care for a malaria episode have no documented evidence of such an episode in their record, despite being under hospital supervision. This deficiency can have substantial therapeutic implications. It is clear that proper recording of malaria cases in primary care is essential and can be improved on a par with other countries [29].

Imported malaria is increasing in non-endemic areas due to the rise of international travel, migratory flows and, probably, other unknown factors [30]. This finding indicates significant under-recording of malaria cases in primary care settings, potentially leading to delays in diagnosis and treatment as well as hindering the monitoring of patients' health and follow-up care. It is also very interesting to note the low vaccination rates against hepatitis A, hepatitis B, or typhoid fever among the cases studied. This circumstance may warrant a separate analysis, as described in the literature [31].

These findings emphasize the need for improved record-keeping practices in primary care, especially concerning infectious diseases like malaria and HIV. Establishing clear protocols and guidelines for documenting such cases is essential to ensure timely and appropriate care and to facilitate effective communication between primary care providers and specialists. Additionally, enhancing healthcare provider education and awareness as to the significance of accurate record-keeping may help mitigate these issues and ultimately improve patient outcomes.

The results unequivocally highlight significant concerns regarding the documentation of malaria episodes and travel history in the PCMR. The low rates of recorded malaria cases and intent to travel, particularly for the vulnerable VFR population, highlight the need for improved surveillance and educational initiatives in primary care. These actions can aid in identifying high-risk areas and providing targeted interventions to reduce malaria incidence and enhance patient care.

### Clinical management and suboptimal follow-up of malaria in primary care

The findings of this study reveal a worrisome trend in the adherence with malaria case management guidelines among PCPs in the study area. Adherence was notably low in the sample, indicating a significant gap in the implementation of recommended protocols for managing malaria cases. This shortfall in adherence underscores the need for targeted interventions and educational initiatives aimed at improving the adherence with established guidelines, ultimately enhancing the quality of malaria care provided in primary care settings. Addressing this issue is of utmost importance to effectively combat malaria and reduce its impact on public health.

The findings indicate sub-optimal follow-up of malaria patients in primary care settings. Among the 90 patients with a documented episode in their primary care records, only 40% had recorded follow-up in the clinical history. The remaining 60% had no such record of follow-up, suggesting a significant gap in the continuity of care for these individuals. This deficiency may have long-term health implications and underscores the need for healthcare systems to enhance the monitoring and management of malaria cases in primary care.

### Records of previous episodes of malaria

Investigating whether the existence of a previous malaria episode contributes to better preparedness for subsequent encounters with the disease raises a significant question. Future studies may explore this potential correlation, shedding light on whether prior exposure enhances preventive measures and management strategies, ultimately improving patient outcomes.

### Developing a comprehensive public health action plan

Building upon the findings presented above, there is a compelling case for devising a comprehensive public or community health action plan aimed at monitoring, controlling, or analysing “hotspot” areas with a higher concentration of patients at increased risk. This strategic approach would facilitate targeted educational initiatives and other interventions, particularly focusing on VFR travellers. To achieve this, several solutions are proposed that can be implemented in health centres and outpatients clinics (Table 5).

**Table 5:** Innovative strategies for enhancing care in health centers and outpatients clinics

Strategy	Description	Implementation example
Promotional Posters	Visually engaging posters in health centers and clinics highlighting the importance of pre-travel consultations	Posters in waiting rooms and consultation areas
Digital Media Campaigns	Use of social media, websites, and other digital platforms to spread awareness about travel health	Regular updates on health center social media pages
Educational Workshops	Organizing workshops or seminars on travel health for patients, focusing on prevention and the importance of pre-travel advice	Monthly workshops hosted by healthcare professionals
Collaborations with Travel Agencies	Partnering with travel agencies to provide health-related information and promote pre-travel health consultations	Informational brochures distributed by travel agencies
Reminder Services	Implementing reminder services via SMS or email to encourage patients to consider travel health consultations	Automated reminders sent to patients with upcoming international travel bookings
In-Clinic Health Promotion Teams	Dedicated staff or volunteers in clinics to inform and encourage patients about travel health during their visit	Health promotion volunteers engaging with patients in waiting areas

Such a plan could involve the identification of areas where malaria cases are concentrated, potentially utilizing geospatial mapping techniques as demonstrated in this study (Fig. 2). By pinpointing these high-risk zones, healthcare authorities and providers could implement tailored educational campaigns, distribute preventive resources, and strengthen surveillance efforts in these specific regions. Additionally, the plan could include strategies to improve healthcare record-keeping and documentation practices in primary care, addressing the current issue of underreporting or lack of documentation for malaria and other health conditions.

## Limitations

The study spans a lengthy period of time (2006–2023), which may influence the results obtained, as the number of cases is insufficient to carry out a study in stages. Vaccination data obtained from medical records should be interpreted with caution due to a potential bias caused by the lack of a record of vaccinations administered at the hospital or TM centres. It is further acknowledged that the study lacks data on how many patients initially sought care in primary care before visiting the hospital. Although it is hypothesized that this number is quite low, further investigation is needed to comprehensively assess cases where malaria was not suspected or a misdiagnosis occurred. Additionally, not all elements of malaria prophylaxis/prevention were considered in the study; such as stand-by emergency treatment [32].

The development of a targeted public or community health action plan informed by clinical and geospatial data and focused on high-risk areas and specific patient groups can significantly enhance malaria control efforts and contribute to improved healthcare practices in primary care settings.

## Conclusions

This study underscores the critical importance of equipping PCPs with the knowledge and skills necessary to proficiently manage malaria cases, particularly in regions where malaria is not endemic. This need becomes even more pronounced among early-career PCPs. Enhancing their preparedness to offer precise guidance and advice to travellers has significant implications, as the effectiveness of PCP interventions directly mitigates the impact of malaria, even in non-endemic regions. Comprehensive training and support for PCPs in primary care settings can play a pivotal role in achieving this goal and ensure that travellers receive the appropriate care and information necessary to prevent infection.

## Data Availability

M.L.R. has full access to and is the guarantor for the study data. The datasets generated are available from the corresponding author on reasonable request.

## Abbreviations

HC: Health centre
PCMR: Primary care medical records
PCP: Primary care physicians
TM: Travel medicine
VFR: Visiting friends and relatives
